# Professor Tiedemann

**Published:** 1854-07

**Authors:** 


### PROFESSOR TIEDEMANN.

The name of this distinguished Anatomist and Physiologist is familiar to all medical readers. It is grateful to read of such marks of respect as are shown him by his countrymen. Such homage repays one for a life-time of toil. The *Gazette Hebdomadaire* says:—

The *Fête Jubilaire* of the celebrated anatomist, Frederick Tiedemann, who was made Doctor of Medicine in March, 1804, and who has resided at Frankfort four years, has just been celebrated in that town. He was Professor at Heidelberg more than forty years, and he retired from the University in 1849, in consequence of a great domestic affliction—namely, when his son was shot by the Prussian soldiers. The Universities of Heidelberg, of Giessen, of Friburg, and of Wurtzburg; the Imperial Society of Naturalists and of the Academy of Sciences at Munich; the Medical Corporation of Mayence; the Learned Societies of Frankfort, and the Municipality of the town of Heidelberg, sent envoys to give him their felicitations. The Society of Natural History of Frankfort opened a subscription for a gold medal, executed at Munich, to be presented to this celebrated Anatomist. On one side is a portrait of the Professor, and on the other a star-fish, the subject of his first great monograph, which was rewarded by the Institute of France.

